# Low-temperature radiofrequency coblation reduces treatment interval and post-operative pain of laryngotracheal recurrent respiratory papillomatosis

**DOI:** 10.1042/BSR20192005

**Published:** 2020-05-26

**Authors:** Fang Hao, Liyan Yue, Xiaoyan Yin, Xiaotong Wang, Chunguang Shan

**Affiliations:** Department of Otolaryngology, The Second Affiliated Hospital of Hebei Medical University, Shijiazhuang 050000, Hebei, China

**Keywords:** human papilloma virus, laryngotracheal recurrent respiratory papillomatosis, larynx, laser vaporization, Low-temperature radiofrequency coblation

## Abstract

Laryngeal papillomatosis is a benign disease in the larynx but with the potential to develop into significant complications as a result of its high recurrence rate. CO_2_ laser and radiofrequency controlled ablation (coblation) have been used to treat recurrent respiratory papillomatosis, but detailed comparisons of their respective treatment outcomes are not fully investigated. This retrospective study examines the procedure time, time interval between interventions, blood loss during operation, post-operative complications and pain scores among patients who received either CO_2_ laser or radiofrequency coblation interventions for laryngotracheal recurrent respiratory papillomatosis. Compared with CO_2_ laser intervention, radiofrequency coblation significantly reduced operation time, time interval between interventions, blood loss during operation and number of times bipolar electrocoagulation needed in each procedure. Post-operatively, pain scores after radiofrequency coblation were significantly lower than those after CO_2_ laser intervention. Incidence rates of post-operative complications, in terms of palate pharyngeal mucosa damage, bleeding and subcutaneous emphysema, were also significantly reduced after radiofrequency coblation. Low-temperature radiofrequency coblation is a superior intervention compared with CO_2_ laser against laryngotracheal recurrent respiratory papillomatosis.

## Introduction

Laryngeal papillomatosis is a benign disease in the larynx but with the potential to develop into significant complications as a result of its high recurrence rate. In the United States, it is estimated that the incidence rate of laryngeal papillomatosis is 4.3 cases per 100000 population in children [1], which is also a common cause of airway obstruction, aphonia and hoarseness among paediatric patients. Laryngeal papillomatosis cases are often recurrent, and its recurrence is more frequent among children than adults. Although this disease usually becomes quiescent when reaching adulthood, children often need multiple surgical procedures to control symptoms at young age [2].

In 1990, the human papilloma virus (HPV) was identified as the aetiological agent responsible for laryngotracheal papillomatosis, and HPV type 6 and type 11 in particular cause benign airway papillomata. Incidents of recurrent respiratory papillomatosis are usually unpredictable and recurrent, and frequently show resistance to treatments [2]. Although various medical and surgical approaches have been developed to treat recurrent respiratory papillomatosis, there have been few studies comparing efficacy of different treatments [1]. To date, CO_2_ laser is still the preferred technique to treat laryngeal papillomata in many Chinese hospitals, and laryngeal microdebrider has become more popular due to shorter procedure time and less chance of scarring [3].

Recently, radiofrequency controlled ablation (coblation) has been increasingly performed in otolaryngological procedures [4–6]. The first application of radiofrequency coblation was reported in 2003 for the resection of tracheal recurrent respiratory papillomatosis [7]. The non-thermally disruptive property, as well as precision, makes radiofrequency coblation an increasingly popular therapy for laryngotracheal recurrent respiratory papillomatosis.

In this retrospective study, we examined the procedure time, time interval between interventions, blood loss during operation, post-operative complications and pain scores among patients who received either CO_2_ laser or radiofrequency coblation treatments for laryngotracheal recurrent respiratory papillomatosis.

## Materials and methods

### Patients

All patients who met the following inclusion criteria were recruited: (1) with laryngotracheal recurrent respiratory papillomatosis; (2) treated with either CO_2_ laser or radiofrequency coblation for a minimum of 5 years. The time interval between surgical interventions required for symptomatic control was determined, after either CO_2_ laser or coblation interventions, respectively.

### Coblation procedure

Briefly, patients were put under general anaesthesia. The zero-eight Hopkins rod endoscope and camera were employed, together with the Storz C laryngoscope (Storz, Tuttlingen, Germany) to visualise the tracheobronchial lesion. The tip of an EICA7070-01 PROCISE LW wand (Arthrocare, U.S.A.) was used in the coblation with power level set at 800–1200 J. The tip of the wand was placed on the lesion at a 90-degree angle to minimise damage to adjacent tissues.

### CO_2_ laser procedure

Briefly, patients were put under general anaesthesia. A 1.27-cm 3M tape was used to carefully wound 5.1 cm up the endotracheal tube balloon. Moist cottonoids attached to long strings were placed in the subglottic space for protection. Laryngoscopy was performed with a laryngoscope using a tooth guard. A model 450 Coherent CO_2_ laser was used to vaporise the papillomas, controlled by a micromanipulator attached to a Zeiss operating microscope.

### Blood loss during operation

Blood loss in operation was assessed by swab weighing as previously reported [8]. Briefly, the swabs were weighed as soon as possible after contamination with blood so that the loss by evaporation is minimised. In the case of uncontrollable bleeding, bipolar electrocoagulation was performed, and the number of times recorded for each surgery.

### Post-operative complications and pain scores

All patients were observed overnight after the procedure, complications were treated accordingly and subsequently recorded. The next morning before being discharged, pain severity of all patients were assessed using the visual analogue scales (VAS), where patients were instructed to indicate their pain severity by marking a scale from 0 to 10 (0 for ‘no pain at all’ and 10 for ‘maximal pain’). Pain scores were categorised into after CO_2_ laser or after radiofrequency coblation, respectively.

### Statistical analysis

Statistical analysis was performed using SPSS software (SPSS Inc., U.S.A.). Values are mean ± standard deviation (SD). Normality of data distribution was tested using the Kolmogorov–Smirnov test, and Student’s *t* test and chi-square test were used to analyse data significance for normally and non-normally distributed data, where appropriate. *P*<0.05 was considered as statistically significant.

## Results

We retrieved patient records in the Second Affiliated Hospital of Hebei Medical University, and patients fitting the following criteria were analysed in the current study: (1) with laryngotracheal recurrent respiratory papillomatosis and full recorded timeline of receiving either CO_2_ laser or radiofrequency coblation for a minimum of 5 years; (2) age > 25 years; (3) with operative records of blood loss and bipolar electrocoagulation; (4) with records of post-operative complications and pain scores. As shown in Table 1, a total of 41 patient records fulfiled the above criteria, of which 19 were males and 22 were females. Among all 41 patients, 241 CO_2_ laser interventions and 68 radiofrequency coblation interventions were performed.

**Table 1:** Patient characteristics

Parameters	
**Total number**	41
**Gender (male/female)**	19/22
**Age (years)**	38.6 ± 6.1
**Times of procedures (*n*)**	
**Laser**	241
**Coblation**	68

*n* indicates number of times each procedure was performed in all patients. Values are mean ± SD.

**Figure 1:**
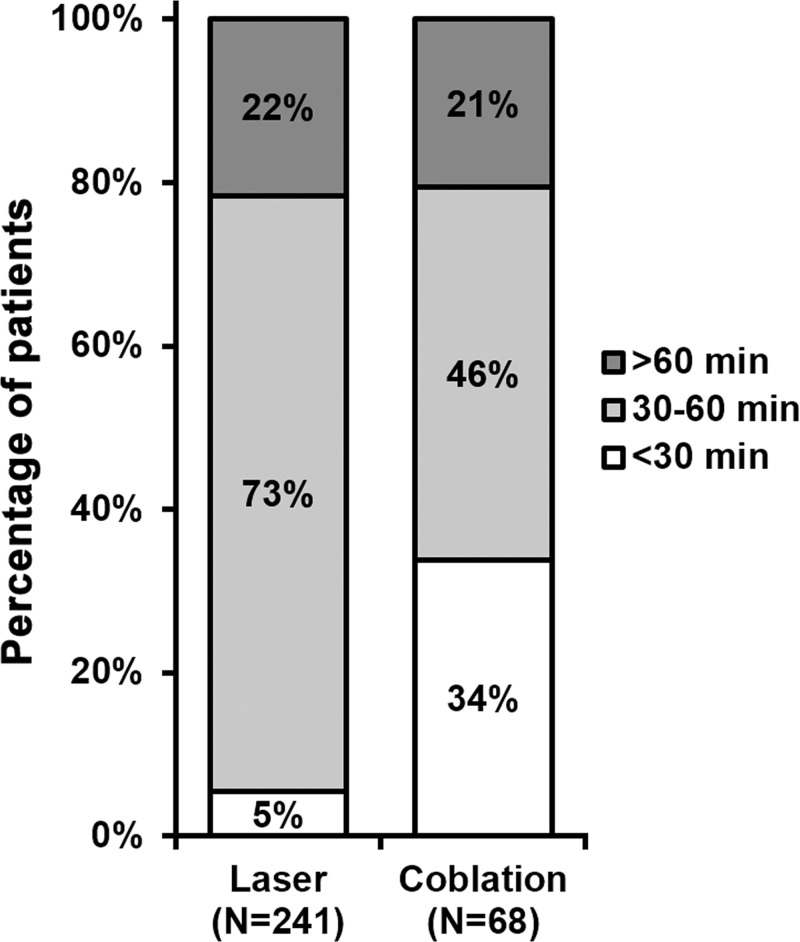
Operation time of each procedure *n* indicates number of times each procedure was performed in all patients. Percentages do not sum up to 100% due to rounding.

**Figure 2:**
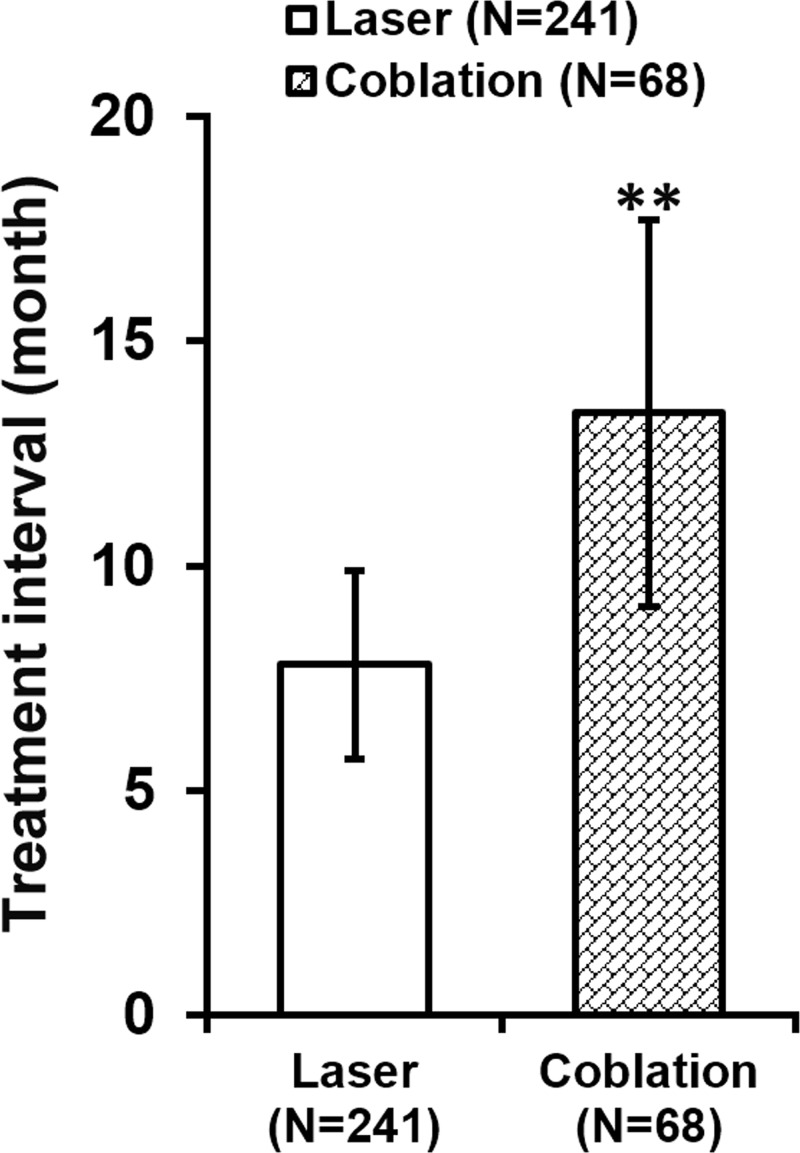
Time interval after CO_2_ laser or radiofrequency coblation before the next intervention *n* indicates number of times each procedure was performed in all patients. Values are mean ± SD. **P<0.01.

Upon examining their records, we found that percentages of operation time over 60 min were comparable between the two types of interventions (Figure 1). On the other hand, majority (73%) of CO_2_ laser took 30–60 min to complete compared with 46% for coblation procedure. Notably, 34% coblation procedures were completed within 30 min, markedly lower than 5% for CO_2_ laser of the same operation time category. Furthermore, the median time length of coblation procedure was 39 min (range: 22–69 min), while the median time length of CO_2_ laser was 51 min (range: 25–74 min) (Table 2). In addition, the time interval between surgical interventions required for symptomatic control was determined. As shown in Figure 2, the time interval after CO_2_ laser was significantly shorter than that after coblation intervention.

**Table 2:** Median time length of procedures

	Laser (*n*=241)	Coblation (*n*=68)
**Median time length (min)**	51	39*
**Range of time length (min)**	25–74	22–69

* *P*<0.05.

**Figure 3:**
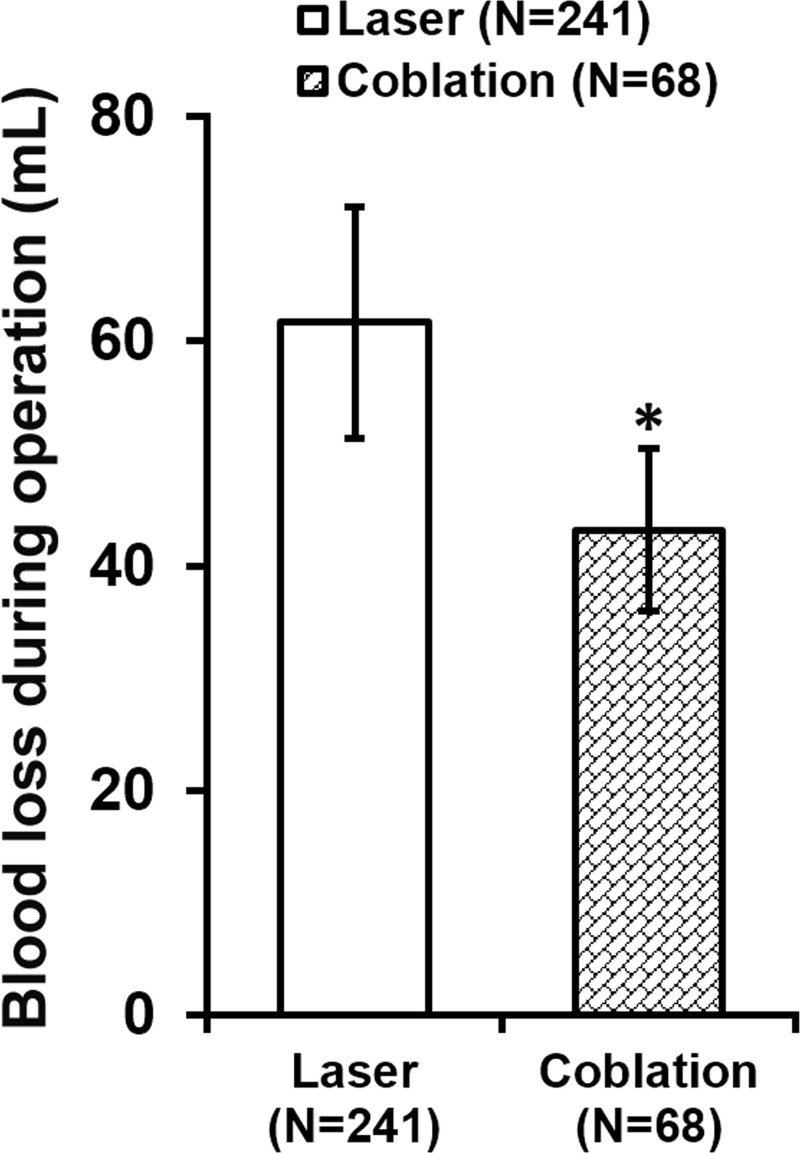
Blood loss during operation *n* indicates number of times each procedure was performed in all patients. Values are mean ± SD. *P<0.05.

**Figure 4:**
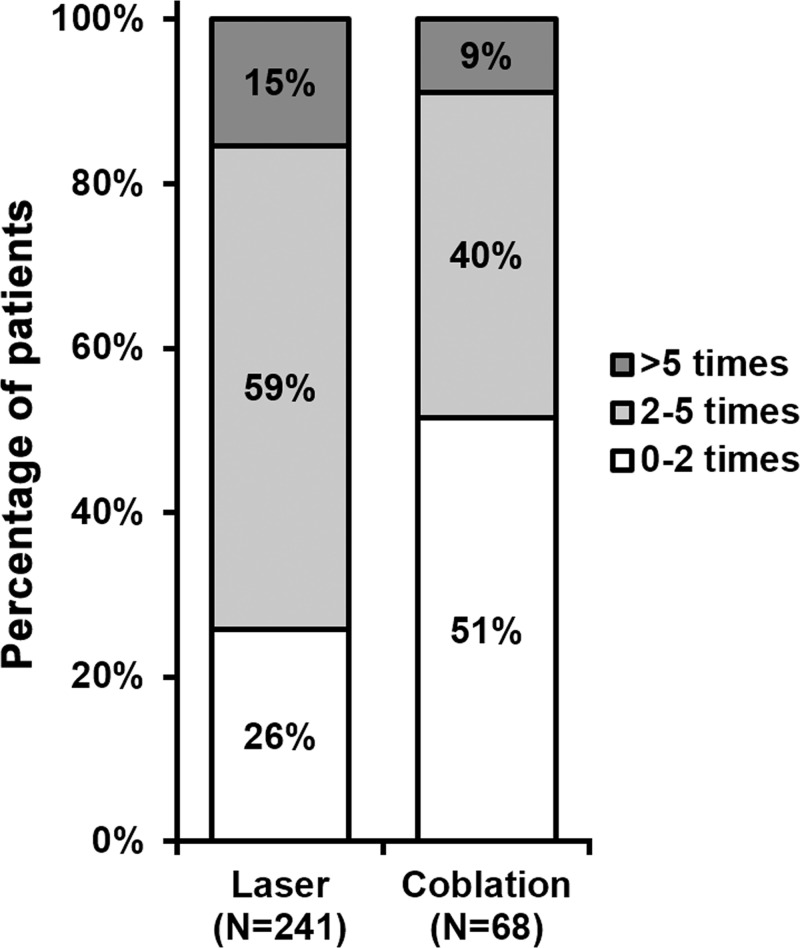
Number of times bipolar electrocoagulation used in each procedure *n* indicates number of times each procedure was performed in all patients. Percentages do not sum up to 100% due to rounding.

During each procedure, amount of blood loss was measured by swab weighing, and we found that on average, coblation significantly reduced the blood loss compared with CO_2_ laser (Figure 3). Furthermore, the number of times bipolar electrocoagulation was performed to control bleeding during operation was also analysed, where bipolar electrocoagulation was used much less frequently during coblation than during CO_2_ laser (Figure 4).

**Figure 5:**
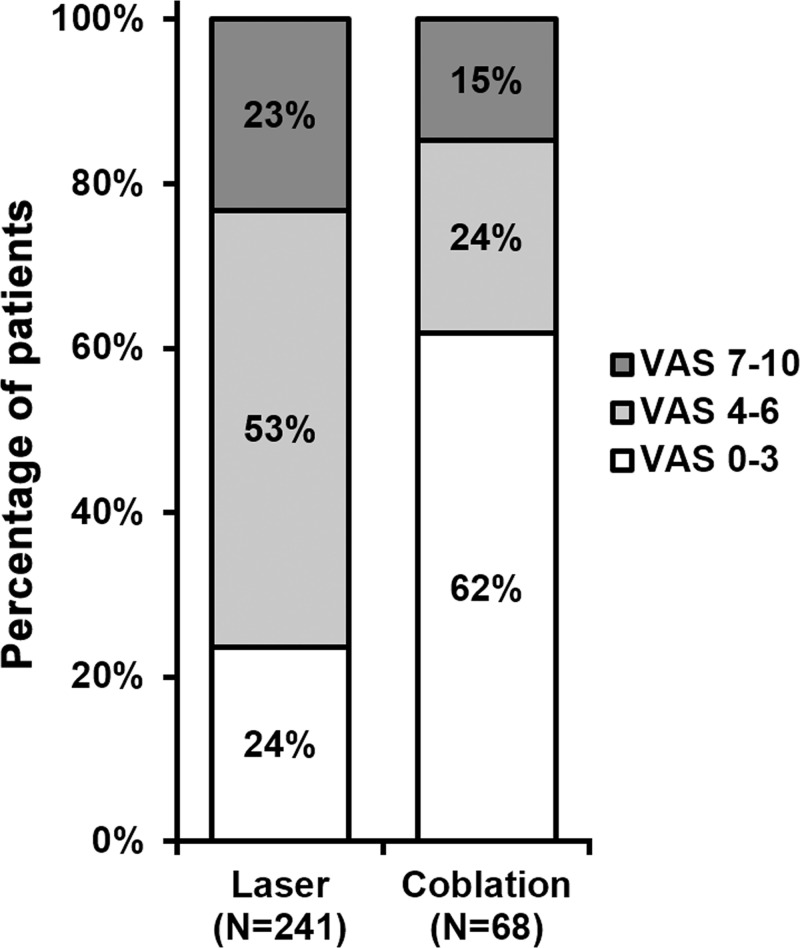
Post-operative pain scores *n* indicates number of times each procedure was performed in all patients. Percentages do not sum up to 100% due to rounding.

After completion of each operation, patients were monitored to record their complications (Table 3). Incidence rates of palate pharyngeal mucosa damage, bleeding and subcutaneous emphysema were significantly lowered after coblation compared with after CO_2_ laser, while incidence rates of vocal cord adhesion, laryngostenosis and asphyxia were indistinguishable between the two interventions. After each procedure and before being discharged, post-operative pain of all patients was evaluated by VAS scores (Figure 5), which exhibited categorically reduced VAS scores following coblation as compared with following CO_2_ laser.

**Table 3:** Post-operative complications after each procedure

Complications [% (*n*)]	Laser (*n*=241)	Coblation (*n*=68)
**Palate pharyngeal mucosa damage**	22.0% (53)	11.7% (8)*
**Bleeding**	12.4% (30)	5.9% (4)*
**Subcutaneous emphysema**	6.6% (16)	4.4% (3)*
**Vocal cord adhesion**	3.7% (9)	4.4% (3)
**Laryngostenosis**	2.9% (7)	2.9% (2)
**Asphyxia**	0.8% (2)	1.4% (1)

* *P*<0.05.

## Discussion

To date, CO_2_ laser, microdebrider and radiofrequency coblation are the most commonly performed surgeries to treat recurrent respiratory papillomatosis. Laser power results in thermal vaporisation of water, causing disruption of cells, where DNA of HPV could be detected in the ing plume [9]. Such dispersion of HPV DNA has been speculated to give rise to distal seeding of lesions into the tracheobronchial tree in 13% of patients according to a previous study [3], causing potential surgical complications that are more demanding on both the anaesthetist and the otolaryngologist. These distal lesions often require a laser bronchoscope to treat, which produces a beam tangential to the lesions, potentially causing higher risk of collateral damage and further distal seeding. Laryngeal microdebrider, on the other hand, is able to rapidly control recurrent respiratory papillomatosis locally, but requires more surgical procedures [10] and causes more intra-operative bleeding [11] than CO_2_ laser. Although bleeding caused by microdebrider can be kept under control using topical adrenaline in limited laryngeal diseases [12], it is nevertheless a major surgical complication in gross tracheal diseases, which further limits the amount of treatable diseases with a single procedure. However, both CO_2_ laser and microdebrider approaches have clinical difficulties. CO_2_ laser intervention requires a team of fully trained surgical personnel, during which a laser tube may also be needed that further limits surgical access. While surgery using microdebrider could be hindered by more frequent bleeding, which obstructs the view and may compromise lesion removal.

As compared, radiofrequency coblation offers significant advantage over the other two surgical approaches in treating recurrent respiratory papillomatosis, as suggested by previous studies and our current one. For instance, radiofrequency coblation has been successfully performed on patients with extensive laryngeal papillomata and a paediatric laryngeal papillomatosis patient, with minimal damage to underlying tissues and a bloodless field [13,14]. To bring further evidences supporting the benefits of radiofrequency coblation to treat recurrent respiratory papillomatosis, our study has demonstrated that operation time, time interval between interventions, blood loss during operation and number of times bipolar electrocoagulation needed were all significantly reduced. Even post-operative complication and pain were found to be much improved after radiofrequency coblation as compared with CO_2_ laser. Therefore, our data have further indicated that low-temperature radiofrequency coblation is a superior intervention compared with CO_2_ laser against laryngotracheal recurrent respiratory papillomatosis.

The above observed advantages of radiofrequency collation make it an ideal clinical treatment against laryngotracheal recurrent respiratory papillomatosis. With this technique, physicians could efficiently and precisely remove papilloma with minimal damage to surrounding tissues hence minimising bleeding, as suggested in our study in terms of reduced blood loss during operation and number of times bipolar electrocoagulation is needed. However, of note, the swab weighing technique to measure blood loss has its own disadvantage affecting either accuracy of measurement or operation time. Because swabs are pre-moisturised with saline, and need to be measured before being used to absorb the blood, which might potentially prolong overall length of the procedure. Alternatively, measuring wet swabs before procedure could avoid unnecessary time, but evaporation of the liquid on the swabs would make the measurement of blood loss relatively less accurate. Other methods, both accurate and quick to perform, to quantitate blood loss during procedure are needed to gain more reliable and reproducible results in evaluating not only blood loss *per se*, but also operation time.

Although earlier retrospective study has compared radiofrequency coblation with that of CO_2_ laser vaporisation for treatment of advanced laryngotracheal recurrent respiratory papillomatosis [7], our current study still holds novelty in the following aspects: (1) larger sample size (41 vs. 6); (2) more comprehensive outcome evaluations (procedure time, time interval between interventions, blood loss during operation, post-operative complications and pain scores vs. interval between treatments only).

## Conclusion

In conclusion, data from our current retrospective study provide strong evidences supporting the efficacy of low-temperature radiofrequency coblation in treating laryngotracheal recurrent respiratory papillomatosis. Our study highlights that, for treatment against laryngotracheal recurrent respiratory papillomatosis, low-temperature radiofrequency coblation is a potentially superior intervention compared with CO_2_ laser.

## Abbreviations

coblation: controlled ablation
HPV: human papilloma virus
VAS: visual analogue scale
